# Re: George *et al.* High seroprevalence of COVID-19 infection in a large slum in South India; what does it tell us about managing a pandemic and beyond?

**DOI:** 10.1017/S0950268821001382

**Published:** 2021-06-28

**Authors:** Carolin Elizabeth George, Leeberk Raja Inbaraj

**Affiliations:** Departmentof Community Health, Bangalore Baptist Hospital, Hebbal, Bengaluru 560024, India

Dear Editor,

Greetings.

We were happy to read the comments about our research Victor Cardenas (HYG-LE-11829). The data were collected between 26 August and 11 September 2020. As the letter states, DJ Halli slum had the least number of cases in the Second wave. We have not conducted similar studies in other poor urban settlements during the first wave, but it is possible that they also had a high seroprevalence, given the impossibility of social distancing, poor mask usage and limited availability of water for handwashing in these settings. India's second wave predominantly affected the middle and upper social class and spared the urban poor. The most plausible explanation for limiting of slum population during the second wave is that the urban poor would have been infected in the first wave. A recent survey done in 24 slums in Bengaluru reported that only 20% had antibodies to the severe acute respiratory syndrome-coronavirus-2 (SARS-CoV-2) [1]. However, the authors have commented about the possibility of an earlier infection, waning of antibodies and the role of T cell immunity in this population. This assumption needs to be further explored in the context of the low infection rate in slums during the second wave.

## References

[1] One in five has antibodies against Covid in slums in Bengaluru: Study. Times of India 2021, May 21. Available at https://timesofindia.indiatimes.com/city/bengaluru/one-in-five-has-antibodies-against-covid-in-slums-in-bengaluru-study/articleshow/82825325.cms (Accessed 27 May 2021).

